# Parental Leave: What Do Physician Societies Provide for Their Employees?

**DOI:** 10.1089/whr.2024.0043

**Published:** 2024-08-12

**Authors:** Sofia von Fedak, Sonya Priven, Amna Khalid, Amanda Brooks, Gregg C. Lund

**Affiliations:** ^1^ University of New England College of Osteopathic Medicine, Biddeford, Maine, USA.; ^2^ Rowan-Virtua School of Osteopathic Medicine, Stratford, New Jersey, USA.; ^3^ Rocky Vista University College of Osteopathic Medicine, Parker, Colorado, USA.; ^4^ Volunteer Mentor, Park City, Utah, USA.

**Keywords:** parental leave, physician societies, workforce policies, parental health benefits

## Abstract

**Introduction:**

Parental leave yields significant health benefits for parents and children. While many medical associations endorse parental leave, it is unknown what parental leave they provide for their employees.

**Objective:**

To assess parental leave policies of national physician societies for their employees including paid versus unpaid and parity between birth mothers and non-birthing parents.

**Methods and Materials:**

A cross-sectional analysis in 2023 examined parental leave policies of national physician societies, including the American Medical Association (AMA), American Osteopathic Association (AOA), and six specialty societies: American College of Obstetricians and Gynecologists (ACOG), American College of Osteopathic Obstetricians and Gynecologists (ACOOG), American Academy of Pediatrics (AAP), American College of Osteopathic Pediatricians (ACOP), American Academy of Family Physicians (AAFP), and American College of Osteopathic Family Physicians (ACOFP). Examination of policies included: duration, whether paid or unpaid; qualifications before receiving benefit; and whether non-birthing, adoptive, and foster parents were covered.

**Results:**

Among the eight societies surveyed, two (25%) did not disclose their policies (ACOG, ACOP), and one (12.5%) lacked a policy (ACOOG). Of the remaining five, two (40%) offered paid leave (AMA, AAP), while three (60%) provided unpaid leave in line with legal requirements (AOA, AAFP, ACOFP). Benefits for non-birthing parents mirrored those for birth mothers, although the AMA offered birth mothers enhanced benefits.

**Conclusions:**

Only a minority of surveyed physician societies provide paid parental leave. Physician societies should consider providing paid parental leave for their employees and making their policies publicly available to promote and model the benefit of paid parental leave.

## Introduction

Parental leave, encompassing paid, and unpaid periods away from work, is a critical employee benefit designed to better allow parents the ability to care for and bond with a newborn or an adopted or fostered child. This benefit has been directly correlated with positive outcomes for parents and their children. Specifically, paid maternity leave is associated with increased breastfeeding,^1,2^ as well as decreased infant mortality rates,^3^ infant and maternal rehospitalization,^4^ levels of depression in mothers,^5^ and child maltreatment.^6^

Even in light of the benefits of paid parental leave, in 2023 these benefits are not universally available in the US.^7^ In a survey of 4,200 human resource professionals at US companies, only 40% provided some form of paid maternity or parental leave.^7^ Government statistics found that 43% of US companies had paid benefits for the birth mother and only 24%–27% provided paid benefits to non-birthing parents.^8,9^

Physician societies advocate for many public health issues, and some have included paid parental leave.^10–12^ Their advocacy, resulted in universal paid parental leave for postgraduate physician trainees.^13^ Professional organizations could only accomplish their strategic goals, including advocacy work, with the support of their employees. However, there is no public data on whether physician societies have translated this into providing paid parental leave as a benefit for their employees. Our study aimed to describe the parental leave policies of a focused group of physician societies for their employees.

## Materials and Methods

The inclusion criteria were: US-based physician societies with a national membership, and either a broad physician organization representing all specialties or a specialty-specific society whose clinical domain is closely connected to families with the greatest exposure to the need for parental leave (obstetrics/gynecology, pediatrics, and family medicine). We included organizations open to both osteopathic physicians (DOs) and allopathic physicians (MDs), as well as those primarily for DOs. Osteopathic-focused organizations were included to evaluate the consistency of society policies with the profession’s stated support for a holistic approach to patient care.^14^

Due to their general roles representing physicians, we included the American Medical Association (AMA) and the American Osteopathic Association (AOA). The American College of Obstetricians and Gynecologists (ACOG), American College of Osteopathic Obstetricians and Gynecologists (ACOOG), American Academy of Pediatrics (AAP), American College of Osteopathic Pediatricians (ACOP), American Academy of Family Physicians (AAFP), and the American College of Osteopathic Family Physicians (ACOFP) were included because these specialties’ patient populations are the most likely affected by parental leave policies.

Requests for each organization’s employee parental leave policy were emailed to each society’s physician and professional leaders. We sent follow-up inquiries if needed. Policies were evaluated for duration of benefit, whether paid (including the percentage of current salary) or unpaid, constraints/qualifications before receiving the benefit, whether non-birthing, adoptive, or foster parents were covered, non-salary benefits and clarification of benefits when two parents were in the same organization. We evaluated each policy twice—once by a member of the investigator group (S.v.F., S.P., A.K.) and the second by the senior author (G.C.L.) Differences in interpretations were reconciled by consensus.

## Results

All eight (100%) organizations responded to the recruitment emails. However, only 5 of 8 (63%) provided parental leave policies as requested (AAP, AAFP, ACOFP, AMA, and AOA). The ACOG stated that it is the organization’s policy to not disclose internal policies. The ACOP confirmed they would not share their policies, but no reason was given. The ACOOG did not have a policy in place for parental leave. There were no conflicts in interpretation between the first and second reviewers.

Of the policies submitted, only AAP and AMA provided paid parental leave to their employees (Table 1). These two societies offer more than the AAP minimum recommended 6–8 weeks of paid leave^10^ at least for the birth mother. The AMA (for the birth mother) provides 8.6 weeks, and the AAP (for all parents) provides 12 weeks of paid leave. AOA, AAFP, and ACOFP do not provide paid parental leave and rather have policies consistent with the 12 weeks of unpaid leave required by the federal Family and Medical Leave Act (FMLA).^15^

**Table 1.** Description of Physician Organization Family Leave Policies for Employees—“Clean”

	Childbirth leave (birth mother)			Non-birthing parent, adoption, or foster parent		
Organization	Duration	Percent of salary	Constraints	Duration	Percent of salary	Constraints
AOA	No paid family leave Up to 12 weeks of unpaid leave	0%	Employed for ≥12 mos and worked ≥1,250 hrs in the most recent 12 mos Must use all paid leave first	No paid family leave Up to 12 weeks of unpaid leave	0%	Employed for ≥12 mos and worked ≥1,250 hrs in the most recent 12 mos Must use all paid leave first
AMA	60 days	90%	Following the start of leave, there is a 7-day waiting period before paid benefits start. During that period, the employee must use all other paid time off	20 days	100%	Following the start of leave, there is a 7-day waiting period before paid benefits start. During that period, the employee must use all other paid time off
ACOOG	No policy	No policy	No policy	No policy	No policy	No policy
ACOG	Undisclosed	Undisclosed	Undisclosed	Undisclosed	Undisclosed	Undisclosed
ACOP	Undisclosed	Undisclosed	Undisclosed	Undisclosed	Undisclosed	Undisclosed
AAP	12 weeks Paid Additional 4 weeks unpaid	6 weeks - 100% 6 weeks - 50%	Scheduled to work at least 20 hrs/week	12 weeks Paid Additional 4 Weeks unpaid	6 weeks - 100% 6 weeks - 50%	Scheduled to work at least 20 hrs/week
ACOFP	No paid family leave Up to 12 weeks of unpaid leave	0%	Employed for ≥12 mos and worked ≥1,250 hrs in the most recent 12 mos Must use all paid leave first	No paid family leave Up to 12 weeks of unpaid leave	0%	Employed for ≥12 mos and worked ≥1,250 hrs in the most recent 12 mos Must use all paid leave first
AAFP	No paid family leave Up to 12 weeks of unpaid leave	0%	Employed for ≥12 mos and worked ≥1,250 hrs in the most recent 12 mos Must use all paid leave first	No paid family leave Up to 12 weeks of unpaid leave	0%	Employed for ≥12 mos and worked ≥1,250 hrs in the most recent 12 mos Must use all paid leave first

Of the policies provided, each society indicated that their policy applied not only to the birth mothers but also to non-birthing, adoptive, and foster parents. The only exception was AMA, which provided longer paid leave for the birth mother (90% of pay for 60 days) than non-birthing parents (100% of pay for 20 days).

Societies that provide unpaid leave consistent with FMLA (AOA, AAFP, and ACOFP) require employees to have been employed for more than 12 months and have worked more than 1,250 hours in the most recent 12 months to be eligible for this benefit. Additionally, employees must use all paid leave (except for up to 5 paid days) before their unpaid leave starts. No vesting period was included in the AAP or AMA policies. The AAP required that the employee must be scheduled to work a minimum of 20 hours/week. No work minimum was noted in the AMA policy. AMA required that other paid leave be used during the 7-day waiting period between applying for and receiving paid parental benefits.

## Discussion

Of the eight surveyed physician societies, only 2 (25%) (AAP and AMA) documented providing their employees with paid parental leave. Both of these societies’ benefits surpass the AAP-recommended minimum paid leave for birth mothers, but only the AAP policy benefits surpass this level for non-birthing parents.^10^ For all organizations, with one exception, the same parental leave (paid or unpaid) provided to the birth mother was also provided to non-birthing, adoptive, and foster parents.

Research demonstrates that paid family leave policies benefit employees and their families’ lives and well-being. Referencing these and other data physician societies such as the AAP have publicly voiced support for paid parental leave.^10–12^ However, except for the AMA and AAP, other organizations do not provide these benefits to their employees.

Our findings for the surveyed physician societies seem consistent with the paid parental leave provided at ∼27%–40% of private US companies.^7,8^ There are wide variations in US companies’ benefits to birth mothers versus non-birthing parents. For birth mothers in 2023, a congressional research report of private employers shows that 43% of companies provide short-term disability insurance that can be used for pregnancy, but this is only available to the birth mother.^9^ In the same report, only 24% provided these benefits to non-birthing parents. However, there is wide variation in the provision of both short-term disability benefits (19%–74%) and benefits for non-birthing parents (10%–51%), depending on the industry.

While there is no specific data on paid parental leave in physician societies, there is data on three groups in medicine: medical students, postgraduate trainees, and employed physicians. Kraus et al. reviewed the parental leave policies online at 199 US MD and DO granting medical schools.^16^ Only 65 (32.6%) institutions had publicly available parental leave policies. This report did not differentiate between paid and unpaid leave. Most of the policies, 38 (58.3%), provided benefits to both the birth mother and non-birthing parents.

As of 2022, all Accreditation Council for Graduate Medical Education (ACGME) accredited post-graduate physician training programs must provide a minimum of 6 weeks of parental leave at 100% pay.^13^ In a study of parental leave policies of graduate medical education (GME) programs associated with the top 50 MD-granting medical schools before the ACGME mandate, 42% of 59 schools had paid parental leave policies, averaging 5.1 weeks of leave. An earlier literature review of GME policies found a wide variation of programs offering parental leave, ranging from 22% to 90%. In this review, they also did not differentiate between paid and unpaid leave.^17^

We evaluated the potential interaction between DO professional philosophy and osteopathic organizations offering paid parental leave. Kraus et al. identified differences in medical school leave policies for students. There was a difference between MD and DO programs, with more DO granting programs having an available policy of 59.0% versus 25.2% of MD programs. However, most DO programs (69.2%) benefit only the birth mother, while 76.9% of MD programs benefit both birth mothers and non-birthing parents.^16^ In our study, none of the osteopathic (general or specialty-specific) organizations provided paid parental leave to their employees, which would not support a relationship between the osteopathic profession’s stated holistic framework of patient care and the benefits they provided to support employee families.

Employed physicians**’** parental leave policy has also been evaluated. A review of parental leave for employed physicians at top US children’s hospitals revealed that the paid parental leave averaged 8 weeks (range 2–12 weeks) with compensation ranging from 50% to 100% of salary covered.^18^ In a similar study of top US hospitals, 78% of the programs provided paid parental leave to employed physicians, with an average duration of 7.9 weeks for the birth mother and 6.6 weeks for the non-birthing parent.^19^ The frequency of paid parental leave for postgraduate trainees and employed physicians describes a pattern where physician employees are provided better benefits than the employee population in general.

Within the organizations we studied, there were other aspects of paid leave besides duration. These included the eligibility requirements of participants. Our results demonstrated that for paid and unpaid parental leave, almost all organizational policies, non-birthing, adoptive, and foster parents have the same benefits as birth mothers. Of the two organizations we studied with paid parental leave, the benefits were for all parents, although the AMA provided longer-duration benefits to birth mothers than to non-birthing parents. These and other characteristics of the organizational policies are included in Table 1.

The documented benefits of paid parental leave and the lack of universal availability reinforce the continued need for advocacy of paid parental leave that could affect not only the employees of physician societies but employees in general.

This project represents an important first step to illuminate real-world support for parental leave, a policy with significant consequences on public health. This preliminary project does have several limitations. We only included a limited sampling of physician societies. Information on policy implementation, other support for those services provided but not included in the leave policy, or support for those providing coverage during another employee’s leave, were not included. These would help better describe the organizational environment. A more comprehensive assessment of parental leave policies is warranted.

Physician organizations leading medicine have an opportunity to further advocate for paid parental leave. Beginning by providing paid parental leave to their employees, but also by making these policies public. Those who provide paid family leave and make their policies public, model support, and challenge other organizations to do the same for their employees.

## Conclusion

The documented benefits of paid parental leave and the lack of universal availability reinforce the continued need for paid parental leave not only the employees of physician societies but employees in general. By providing paid parental leave and making these policies transparent, physician societies can set an example for other institutions and contribute to the broader universal access to paid family leave.

Moving forward, a more comprehensive assessment of parental leave policies across a broader range of organizations is warranted. Such research could provide further insights into the prevalence and characteristics of parental leave policies within the health care sector and inform efforts to promote equitable access to this vital benefit. Ultimately, by inspiring through action and advocacy, physician societies have the opportunity to lead the way in supporting the well-being of their employees and their families.

## Abbreviations Used

AAP: American Academy of Pediatrics
AAFP: American Academy of Family Physicians
ACGME: Accreditation Council for Graduate Medical Education
ACOFP: American College of Osteopathic Family Physicians
ACOG: American College of Obstetricians and Gynecologists
ACOOG: American College of Osteopathic Obstetricians and Gynecologists
ACOP: American College of Osteopathic Pediatricians
AMA: American Medical Association
AOA: American Osteopathic Association
DO: Doctor of Osteopathy
GME: graduate medical education
MD: Medical Doctor
